# Supplementary material for: Identification of photoperiod-induced specific miRNAs in the adrenal glands of Sunite sheep (Ovis aries)
Source: Front Vet Sci. 2022 Jul 22;9:888207. doi: 10.3389/fvets.2022.888207 (PMC9354845; doi:10.3389/fvets.2022.888207)
Supplement: Supplementary file 1 [file Data_Sheet_1.ZIP › Supplementary file/miRDeep2 file.zip!/miRDeep2 file/Know_Structure/SP42A3/oar-mir-487b.pdf]

miRBase precursor : oar-mir-487b
Total read count : 1944
oar-miR-487b-5p read count : 32
oar-miR-487b-3p read count : 1912
remaining reads : 0

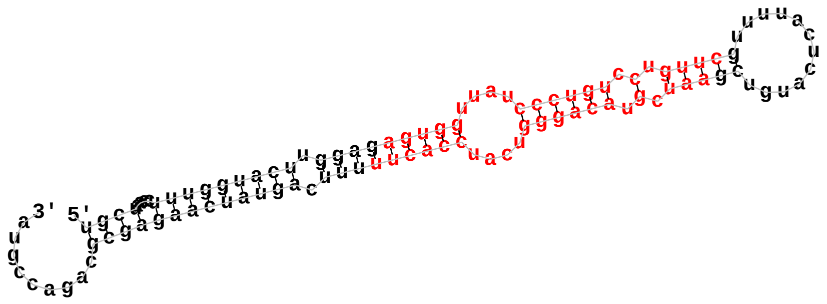

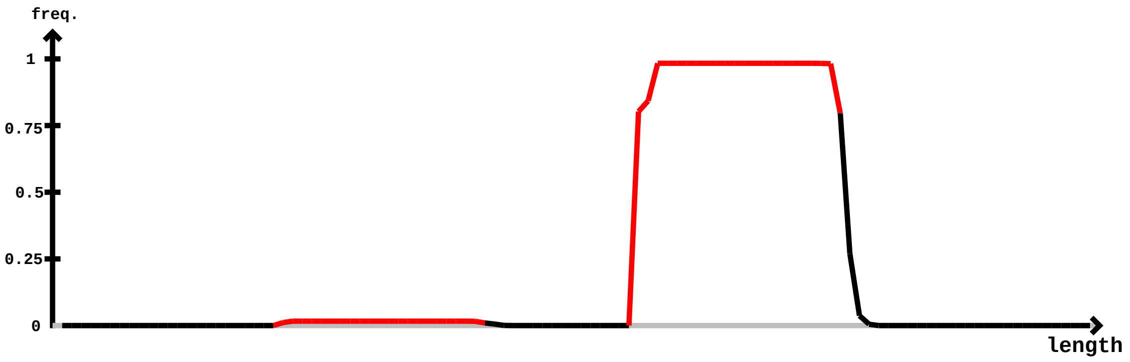


```
                                                                oar-miR-487b-3p
                     oar-miR-487b-5p
5'- ugcuaaccuuugguacuuggagagugguuaucccuguccuguucguuuuacucaugucgaaucguacagggucauccacuuuuucaguaucaagagcgcagaccgua   -3'    exp
    .(((....(((((((((.(((((((((....((((((.(.(((((............))))).).))))))....))))))))).))))))))))))..........    reads      mm        sample
    ......................agugguuaucccuguccugu.................................................................    1          0         seq
    ......................agugguuaucccuguccuguu................................................................    11         0         seq
    ......................agugguuaucccuguccuguuc...............................................................    5          0         seq
    ......................agugguuaucccuguccuguucg..............................................................    4          0         seq
    ......................agugguuaucccuguccuguucgu.............................................................    1          0         seq
    .......................gugguuaucccuguccuguu................................................................    1          0         seq
    .......................gugguuaucccuguccuguuc...............................................................    3          0         seq
    .......................gugguuaucccuguccuguucg..............................................................    6          0         seq
    ...........................................................aaucguacagggucaucca.............................    1          0         seq
    ...........................................................aaucguacagggucauccac............................    2          0         seq
    ...........................................................aaucguacagggucauccacu...........................    356        0         seq
    ...........................................................aaucguacagggucauccacuu..........................    983        0         seq
    ...........................................................aaucguacagggucauccacuuu.........................    217        0         seq
    ...........................................................aaucguacagggucauccacuuuu........................    1          0         seq
    ............................................................aucguacagggucauccacu...........................    5          0         seq
    ............................................................aucguacagggucauccacuu..........................    28         0         seq
    ............................................................aucguacagggucauccacuuu.........................    40         0         seq
    ............................................................aucguacagggucauccacuuuu........................    5          0         seq
    .............................................................ucguacagggucauccacu...........................    1          0         seq
    .............................................................ucguacagggucauccacuu..........................    12         0         seq
    .............................................................ucguacagggucauccacuuu.........................    195        0         seq
    .............................................................ucguacagggucauccacuuuu........................    56         0         seq
    .............................................................ucguacagggucauccacuuuuu.......................    10         0         seq
```
